# Web-based metabolic network visualization with a zooming user interface

**DOI:** 10.1186/1471-2105-12-176

**Published:** 2011-05-19

**Authors:** Mario Latendresse, Peter D Karp

**Affiliations:** 1 Bioinformatics Research Group, SRI International, 333 Ravenswood Ave, Menlo Park, CA 94025

## Abstract

**Background:**

Displaying complex metabolic-map diagrams, for Web browsers, and allowing users to interact with them for querying and overlaying expression data over them is challenging.

**Description:**

We present a Web-based metabolic-map diagram, which can be interactively explored by the user, called the *Cellular Overview*. The main characteristic of this application is the zooming user interface enabling the user to focus on appropriate granularities of the network at will. Various searching commands are available to visually highlight sets of reactions, pathways, enzymes, metabolites, and so on. Expression data from single or multiple experiments can be overlaid on the diagram, which we call the Omics Viewer capability. The application provides Web services to highlight the diagram and to invoke the *Omics Viewer*. This application is entirely written in JavaScript for the client browsers and connect to a Pathway Tools Web server to retrieve data and diagrams. It uses the OpenLayers library to display tiled diagrams.

**Conclusions:**

This new online tool is capable of displaying large and complex metabolic-map diagrams in a very interactive manner. This application is available as part of the Pathway Tools software that powers multiple metabolic databases including Biocyc.org: The Cellular Overview is accessible under the Tools menu.

## Background

Web-based applications using a zoomable user interface (ZUI) are becoming a familiar approach to allowing people to comprehend complex information spaces. Examples include zoomable genome browsers [1,2] and Google Maps. The basic user interaction these tools provide are altering the magnification of a large two-dimensional diagram (often with semantic zooming, meaning new details are displayed at higher magnifications), panning, and searching for points of interest.

Metabolic network models are now available for hundreds of organisms due to the high rate of genome sequencing and the availability of software tools for reconstructing metabolic network models from genome sequence information [2-5]. We aim to provide scientists with tools for understanding, exploring, and exploiting metabolic reconstructions.

The Pathway Tools software has had a metabolic network diagram available for a number of years. This diagram has evolved through many forms. It started as a manually assembled diagram for *E. coli *that was available through the desktop version of Pathway Tools only. Later the diagram was generated by an automated layout algorithm, which allowed organism-specific diagrams to be generated for multiple organisms [6]. Zooming was added to the desktop version several years ago, as was the ability to paint large datasets onto the diagram by coloring the nodes and edges of the diagram. The desktop version of the diagram supports a large number of search and highlighting operations, such as finding metabolites by name, finding reactions according to the regulation of their enzymes, and comparing entire metabolic maps. A Web version of the Cellular Overview was created a number of years ago; however, that Web implementation of the diagram was essentially static because of the limitations of early Web technologies: it permitted no searching or zooming, although it would identify metabolites within the diagram on mouseover. The goal of the work reported herein was to modernize the Web version of this diagram, providing search and semantic zoom capabilities that allow users to navigate metabolic networks effectively. The diagram has not changed substantially; its Web implementation has.

This article describes the information content of the Cellular Overview, and our new implementation of the diagram that makes use of modern Web technologies including JavaScript, AJAX, and OpenLayers. This Web-based diagram is now available for BioCyc, and once installed will be available for other Pathway Tools based metabolic databases (DBs) such as YeastCyc (pathway.yeastgenome.org), MouseCyc [7], and AraCyc [8].

## Construction and Content

**Figure 1:**
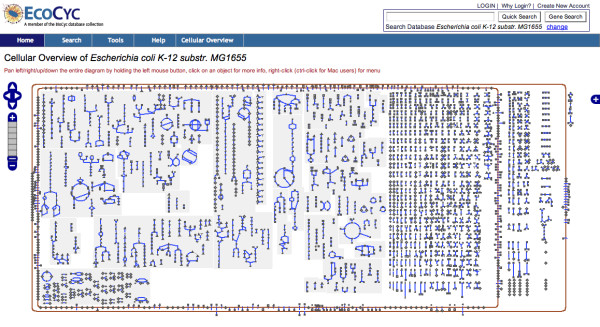
**The Initial Cellular Overview Web Page**. The initial Cellular Overview Web page at http://biocyc.org/overviewsWeb/celOv.shtml for *E. coli *K-12.

The Cellular Overview diagram depicts the biochemical machinery of an organism as described in a PGDB (Pathway/Genome Database) generated by Pathway Tools. Figure 1 shows the initial state of the Cellular Overview for *E. coli *K-12 at the lowest zoom level. Each node in the diagram (such as the small circles and triangles) represents a single metabolite or protein, and each blue line represents a single bioreaction. Within the cytoplasmic membrane, the small-molecule metabolism of the organism is depicted in several regions. The glycolysis and the tricarboxylic acid (TCA) cycle pathways, if present, will be placed in the middle of the diagram to separate predominately catabolic pathways on the right from pathways of anabolism and intermediary metabolism on the left. The majority of pathways operate in the downward direction. Signal transduction pathways, if present, run along the bottom left side of the diagram.

Pathways are grouped into related clusters as indicated by the shaded regions (e.g., amino-acid biosynthesis). The large grid of individual reactions at the right of the diagram represents reactions of small-molecule metabolism that have not been assigned to any pathway. Periplasmic pathways and reactions are shown on the right side in between the two membranes. Transporters and other membrane proteins are embedded in the appropriate membrane. The same metabolite may appear in multiple locations in the diagram because of the high connectivity of the network. Showing all such connections between metabolites would result in so many visible edges as to render the diagram useless.

The Cellular Overview is organism specific, meaning that the pathways, reactions, metabolites, and proteins shown in a version of the diagram for a given organism reflect the biochemistry of that organism as described by its PGDB. For example, 1,000 different incarnations of this diagram are available from the BioCyc.org Web site, reflecting the 1,000 genomes available from BioCyc.

We define multiple semantic zoom levels for the diagram. At higher magnification levels, new information appears in the diagram such as the names of pathways, genes, enzymes, and metabolites.

### Functionality of the Cellular Overview

We give a brief description of the capabilities of the Cellular Overview. The reader is invited to experiment with the diagram by pointing a Web browser to BioCyc.org and selecting the Cellular Overview command under the Tools menu in the top menu bar. Notice that the desired organism should be selected, using the link change in the right-upper corner of the Web page next to the current organism name, before executing the Cellular Overview command. Similarly, selecting a new organism using the change link requires to execute the command Cellular Overview to display the cellular overview of the newly selected organism. Online documentation is available at bioCyc.org under the Help menu, command Website User Guide.

#### Zooming and Panning

Once the Cellular Overview diagram is displayed, the most common operation is to move it left or right (panning), or up or down, since sometimes the entire overview cannot fit in the Web page. This can be done by holding down the left mouse button in a blank area and then moving the mouse in the desired direction. To zoom-in or zoom-out, click the icon in the form of a ladder on the left of the overview Web page. Each step of the ladder is a zoom level. By increasing the zoom level (i.e., going up in the ladder), names of compounds, enzymes, reactions, and pathways are eventually displayed.

#### Searching

In this article, 'searching' and 'highlighting' are synonymous terms. There are several commands to search for reactions, pathways, enzymes, genes, and compounds. The search commands are available from the right-click menu and from the Cellular Overview menu from the top menu bar.

**Figure 2:**
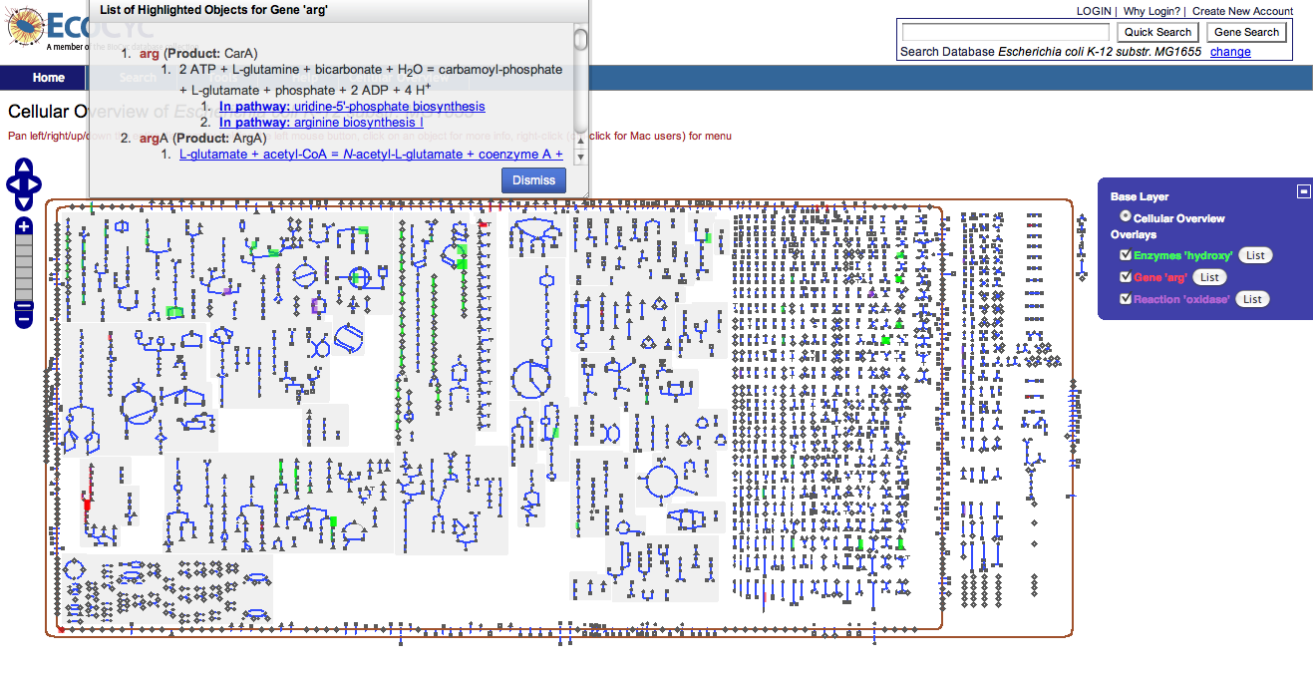
**The Cellular Overview after Three Searches**. The Cellular Overview after three searches. It created three highlighted overlays, one for each search. Highlighting can be activated and deactivated by toggling the radio buttons in the blue Layer Switcher panel on the right. When a 'List' button is clicked, a small window opens up to list the objects found in the overview. Such a window is opened for the list "Gene 'arg' " (the list is long so that the window is scrollable). A gene search finds reactions, RNAs, transporters, and enzymes in the overview. Clicking an item in the list places a marker in the diagram to better locate it.

When a search is done (see Figure 2), the objects found are highlighted in the Cellular Overview diagram using a graphical **overlay**, which is a separate graphical plane in which JavaScript code can draw rectangles, circles, and lines of different colors. The list of overlays is shown in the Layer Switcher panel on the right of the Overview Web page. From this panel you can activate or deactivate (render invisible) specific overlays.

Since each overlay corresponds to a search operation, an overlay is identified with the keyword you entered to do the search. This is the name of the overlay. Next to each name is a button labeled 'List'. Clicking 'List' opens a small dialog window listing the objects found for the corresponding search. Each object name is a hyperlink--clicking any of these links centers the Overview on the corresponding object, and a red marker emphasizes its location.

The searching commands provided are

• **Highlight Pathway(s) **provides two searching mechanisms for pathways in the cellular diagram: by name or frame ID, or by a substring search. The substring search is based on the name, synonyms, and frame ID of the pathways. Highlighting is done on the reaction(s) of the pathway(s) found.

• **Highlight Reaction(s) **provides four searching mechanisms for reactions in the cellular diagram: by name or frame ID, by substring, by EC number, or by enzyme name. The substring search is based on the name, synonyms, and frame ID of the reactions. Highlighting is done on the reaction(s) found.

• **Highlight Gene(s) **provides three searching mechanisms for genes in the cellular diagram: by name or frame ID, by substring, or from a file.

• **Highlight Enzyme(s) **provides two searching mechanisms for enzymes in the cellular diagram: by name or frame ID, or by a substring search. The substring search is based on the name, synonyms, and frame ID of the enzymes. This substring search is identical to the reaction search based on enzymes. Highlighting is done on the reactions and proteins corresponding to the enzyme(s) found.

• **Highlight Compound(s) **provides two searching mechanisms for compounds in the cellular diagram: by name or frame ID, or by a substring search. The substring search is based on the name, synonyms, and frame ID of the compounds. Highlighting is done on the compound(s) found.

#### Tooltips

**Figure 3:**
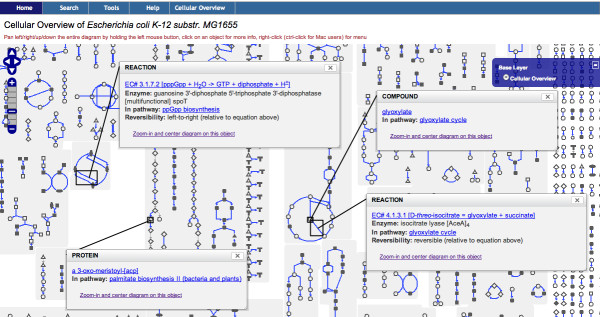
**The Cellular Overview with Four Tooltips Opened**. The Cellular Overview with four tooltips opened. A tooltip opens up by mousing over or by left-clicking an object icon. Tooltips can be repositioned or closed by the user. A tooltip closes automatically unless the user has clicked the button "keep open" (not shown) in the title bar of the tooltip. Web links are also provided in a tooltip to enable the user to display more data about the object or related objects.

Mousing over a Cellular Overview icon (e.g., a 'tee' icon for a tRNA) displays information about the object in a small tooltip popup. Several tooltips can be opened at the same time. Figure 3 shows an example of several opened tooltips.

#### Omics Viewer

In Omics Viewer mode, the Overview is painted with a large dataset for visual analysis of that data in a global metabolic pathway context. Genes (in the case of a gene expression experiment) and proteins (in the case of a proteomics experiment) that are involved in metabolism are mapped to reaction steps in the Cellular Overview, and the range of data values levels in a given experimental dataset is mapped to a spectrum of colors. Reaction steps in the Cellular Overview are colored according to the corresponding data value. Similarly, for metabolomics experiments, compound nodes are colored according to the data value for the corresponding compound. This facility enables the user to see instantly which pathways are active or inactive under some set of experimental conditions.

Expression data is provided by the user in the form of a datafile containing a two-dimensional table of numerical values where each row is labeled with a gene, reaction, compounds, or proteins. The columns are tab delimited. A description of the exact format of the datafile and sample datafiles are provided in the online help accessible via the Omics Viewer panel.

**Figure 4:**
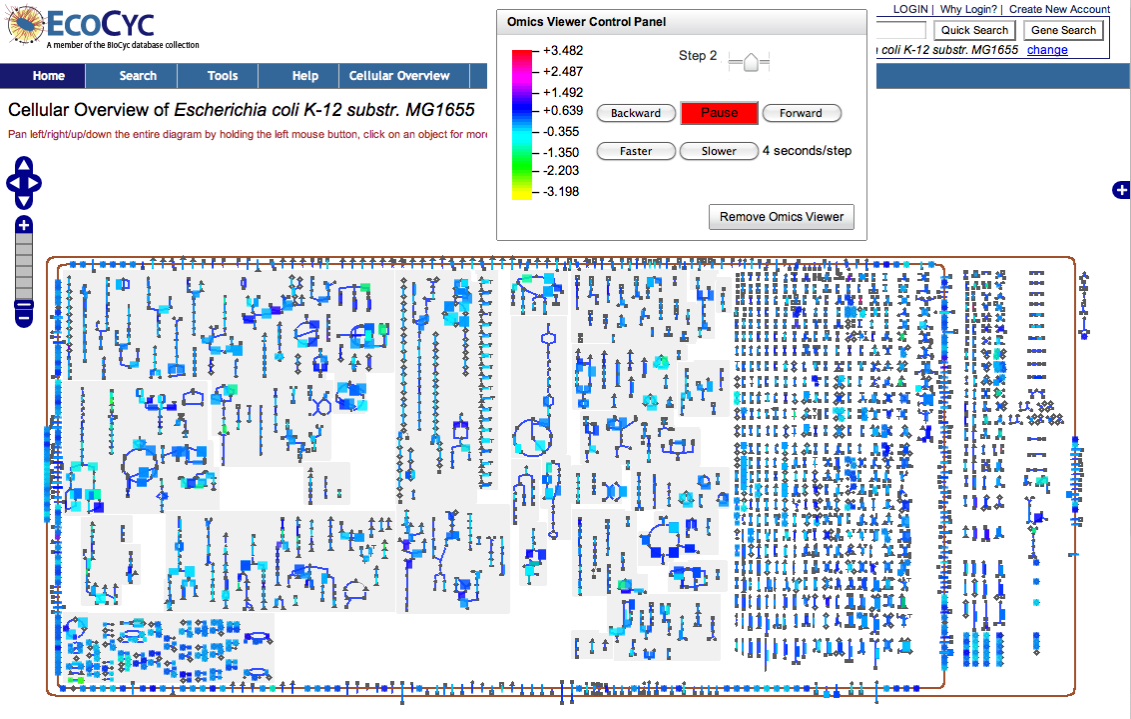
**The Cellular Overview Omics Viewer**. The Cellular Overview with expression data overlaid on metabolic pathways (Omics Viewer). The small window with buttons labeled "Pause", "Slower", and so on, is the control panel. Shown in this example is an animation of three experiments. On the left of the control panel is the color key that describes how data values are mapped to colors. Mousing over a reaction (not shown) displays a tooltip containing data about the reaction and the experimental value for that reaction at the current step of the animation.

Figure 4 shows the Omics Viewer after uploading experimental data for *E. coli *K-12. Essentially all the capabilities of the Cellular Overview are still available in the Omics Viewer, including searching, tooltips, zooming-in, and zooming-out. The user can stop the Omics Viewer animation, go one step forward or backward, and increase or decrease the speed of the animation.

#### Web Services

The Cellular Overview offers a simple form of Web services via HTTP GET requests. Two classes of services are offered: one for highlighting (i.e., searching), the other for invoking the Omics Viewer. A minimal request includes the zoom level and the unique organism identifier. Full documentation for these services is available at http://BioCyc.org/PToolsWebsiteHowto.shtml

An Omics Viewer request specifies a datafile and parameters that influence how the Omics Viewer interprets the data, such as a specification of which columns within the datafile are to be displayed on the Cellular Overview. Two protocols are provided to retrieve the datafile: the http and file protocols.

### Implementation

The Web based Cellular Overview is implemented using mostly the open software OpenLayers (openlayers.org). The Yahoo user interface library (YUI Library) is also used for some of the widget dialogs, tooltips, and asynchronous communication with the server.

The implementation of OpenLayers is entirely done in JavaScript. Since a JavaScript engine is embedded in the popular Web browsers (e.g., IE, Firefox, Safari, Chrome), this implies that no software installation is required to run OpenLayers. The performance of JavaScript engines has steadily increased from 2006 to 2010. Nowadays, JavaScript code is compiled to native code to achieve high performance.

OpenLayers was designed to display geographical road maps by using *tilde *images: for efficiency, large images are sliced into *tiles *and the needed tiles are transfered on demand. Its capabilities are similar to the Google Maps Application Programming Interface (API), but the OpenLayers implementation is open source and their licenses differ greatly. For example, the OpenLayers license does not constrain its usage when used in intranets.

OpenLayers takes care of fetching (and prefetching in some cases) the image tiles required to fill the portion of the view port seen by the user. When the user changes the zoom level, OpenLayers requests the appropriate tiles from the Web server. The coordinates of the tiles, as well as the zoom level, are passed as parameters to the Web server. Additional parameters are specified by our implementation to identify the organism. Then, the entire diagram is reconstructed by OpenLayers by simply laying the tiles contiguously. OpenLayers overlays can be made active, inactive, or deleted by one method call. Overlays are employed by the Cellular Overview to implement Omics Viewer highlighting and highlighting of search results.

#### Base Images

The diagrams for the Cellular Overview can be very large. For example, for some organisms, the diagram can have more than 20,000 pixels in width and height at the highest zoom levels. Such images could not be efficiently transfered from the Web server to a browser. Instead, these images are tiled -- they are sliced in smaller rectangles, the tiles. We use 400 by 200 pixel tiles in the GIF format. The creation of the tiles is done in several steps:

1. A general layout is created for all zoom levels.

2. The exact layouts at specific zoom levels are computed.

3. Based on the layout, one large image with specific colors and text is generated.

4. The large image is sliced into as many tiles as needed.

The first step is done once for each organism, but steps 2 through 4 are done for each zoom level for each organism. The first step places all possible objects in the diagram but the generation at each zoom level might filter out some objects depending on the space available. The computation of step 1 is considered part of the PGDB of the organism.

The second step fixes the exact location of each object in a rectangular coordinate system at a specific zoom level. Some objects are absent at the lower zoom levels. For example, enzymes are not displayed at the lowest zoom level.

The third step generates one bitmap image for each zoom level. This operation is independent from the image format used. From that bitmap image a different type of format (e.g., GIF, PNG) could be generated.

The fourth step takes the entire image and generates each tile as a GIF file. The file system is used as a cache since the tiles are not regenerated unless the PGDB of the organism is modified, which does not occur often.

#### Generating Object Data Location in Images

The generation of GIF files as described in Subsection does not provide any data on the *location *of objects in the image. We call this the *node data*. But this data is needed for tooltips and highlighting--i.e., the result of searching. The tooltips mechanism also needs a textual description of each object in an overview. We call this the *frame data*.

The node and frame data is directly used by the JavaScript code running in the browser. Consequently, the server sends this data to the browser as JavaScript data structures ready to be used by the JavaScript code.

#### Asynchronous Queries

AJAX (Asynchronous JavaScript and XML) is at the core of many interactive Web applications and this implementation uses it throughout. The Cellular Overview uses AJAX for the following operations:

1. For the tooltip mechanism: the Web server is queried for the text data when the mouse stops at any location on the Cellular Overview

2. To retrieve the tiles from the Web server (done by OpenLayers)

3. For all the searches (highlighting): for the auto completion mechanism, the search itself based on strings, names, and the node data to locate the objects on the diagram

4. During the zoom-in and zoom-out operations where the size of the new diagram is required; this might trigger the tiles for that zoom level to be generated by the Web server if the tiles do not exist on the server

#### Omics Viewer

The browser provides the GUI for entering the datafile name and various parameters to control the Omics Viewer. The data is parsed and interpreted by the Web server and a set of objects with their colors is sent back to the browser as JavaScript data. The result can be for a single set of colors if one experiment is requested or multiple sets of colors (i.e., an animation) if several experiments are requested via the datafile and the parameters specified.

Once the JavaScript data is received by the browser, the Cellular Overview creates an OpenLayers layer for each experiment (i.e., each animation step) to do the highlighting. These layers are made active and inactive, by JavaScript code, to create an animation, or if a single experiment is displayed, the sole layer is made active. In summary, no new base diagram is generated, which makes the Omics Viewer very efficient, but overlays are used; once the color data has been transfered to the browser, the animation becomes independent of the Web server, which makes the Omics Viewer efficient.

#### Performance

The Cellular Overview has been optimized to balance work between the server and the browser. The server has all the main data; data transfer is generally postponed until needed. The performance of the Cellular Overview is greatly influenced by the speed of the browser's JavaScript engine. The latest versions of Safari and Chrome (Safari 5.0 and Chrome 6.0) are known to be faster than Firefox 3.6 and Internet Explorer (IE) 7 and 8. Currently, we do not recommend the use of IE 7 or 8 as the other browsers, including IE 9, have a much faster JavaScript engine.

Here are some performance timings for typical operations of the Cellular Overview using Chrome 6.0:

1. First visit to the Cellular Overview, around 4 seconds.

2. Zooming-in or -out without any highlighting, around 2 seconds.

3. Zooming-in or -out with less than 400 highlighted objects, around 4 seconds (this includes the Omics Viewer). The time increases by about 1 second for each 300 objects to highlight.

4. Starting the Omics Viewer with a data file with less than 200 lines, less than 5 seconds. It reaches about 30 seconds with a data file of 5000 lines, which is about the limit of objects that could be named for almost all organisms.

5. Searching for objects, less than 4 seconds for cases resulting in less than 1000 objects.

6. Tooltip, typically 1 second to open.

Notice that all these numbers assume that a server communication occurs; otherwise it is faster, as the browser will be reusing cached data.

## Utility

The Cellular Overview can be used to display the complete set of pathways and biochemical reactions of a single organism. It does not require the installation of special software as it can be accessed with most popular browsers. It enables the user to search and find reactions, proteins, compounds and pathways using various commands. The overview can be displayed at various zoom level with increasing amount of details. It can therefore be useful to navigate the complete reaction networks of an organism with the appropriate level of details. Navigation is made easier by allowing panning in any directions without any constraint.

The Cellular Overview can also be used to display expression data over reactions, compounds and proteins. The expression data can be easily provided by the user via an upload mechanism.

## Discussion

Our discussion focuses mainly in comparing the Cellular Overview to other similar tools available on the Web.

The Pathway Projector (PP) [9] is a Web-based pathway browser that is derived from the KEGG Atlas [10]. It is implemented as a zoomable map using the Google Maps API. Almost the same number of zoom levels are provided: six for the BioCyc Cellular Overview versus five for the Pathway Projector. The Pathway Projector is similar to the Pathway Tools Cellular Overview, but they differ in some major ways:

1. The Cellular Overview generates organism-specific metabolic map diagrams for each organism, whereas the Pathway Projector always uses the same reference metabolic map diagram. The diagram contains all KEGG pathways, but pathways and reactions are whited out on an organism-specific basis. This approach is extremely problematic because the single reference KEGG Atlas pathway image contains far too much information to be comprehensible at low zoom levels -- so why provide them? The first, and perhaps the second, of the five zoom levels in PP convey essentially no information because every graphical element is too small. Even though that reference pathway map diagram contains too much information now, it must grow further over time. Currently, KEGG assigns only half as many biochemical reactions to metabolic pathways as does the MetaCyc DB [11]; MetaCyc in turn is still incomplete with respect to known metabolic pathway knowledge. By generating truly organism-specific metabolic diagrams, Pathway Tools creates diagrams that are more easily comprehensible, do not contain irrelevant pathways, and have space for additional information not found in the KEGG Atlas, such as transporters, and individual reactions not assigned to metabolic pathways.

2. PP visually maps experimental data for genes, enzymes, and metabolites simultaneously onto separate graphical elements, whereas the Cellular Overview does not distinguish genes from enzymes. Although such a distinction could be accomplished via separate time points in an animation.

3. PP diagrams do not show pathway names, enzyme names, or gene names; the Cellular Overview does, at different semantic zoom levels.

4. The Cellular Overview cannot yet search for pathways between compounds whereas the PP can.

5. The Omics Viewer of the Cellular Overview can display an animation of data generated from several experiments by highlighting with key colors. On the other hand, PP displays fixed histograms when processing several experiments and no animation is provided.

The iPath (Interactive Pathways Explorer) [12] Web-based application is an interactive ZUI that like Pathway Projector is based on the KEGG Atlas. As for the Pathway Projector, iPath always uses the same reference pathway image, but with reactions removed on an organism-specific basis; thus, the iPath diagram suffers from the same scalability concerns as does Pathway Projector.

The Reactome Web site [13] has a zoomable pathway map that can be used to navigate to reactions, enzymes, compounds, genes, and pathways. The zoomable interface is not a true ZUI, as the user cannot pan or zoom the map in a fast interactive fashion. Panning can be done only by clicking small arrows that perform a Web server request to send another map shifted in the direction of the arrow clicked. Zooming requires clicking the zoom-in or -out operation and then clicking the map, which always does a Web server request. This interaction is therefore much slower than the Cellular Overview. The diagram is very small and hard to read. No labels appear at higher zoom levels, and no search operations are provided, making it difficult to identify elements of the diagram.

VisANT [14] and Cytoscape [15] both provide zoomable interfaces to cellular networks, but both are based on Java web start, which is slow to load and requires previous download of a Java runtime environment. Furthermore, VisANT and Cytoscape layouts are designed more for protein interaction networks than for metabolic networks, therefore these layouts are hard to interpret for metabolic networks. The work of Klukas and Schreiber [16] provides zooming exploration of metabolic networks, but is not web based. CellPublisher [17] is a Web-based application ZUI that converts hand-drawn CellDesigner [18] diagrams into diagrams that can be published using Google Maps technology. It provides zoomable hand-drawn metabolic network diagrams but does not support analysis of omics data.

MapMan [19] provides a metabolic chart visualization of omics data, but the display is not zoomable, nor does its metabolic chart diagram include pathway diagrams; pathways are depicted as blocks of squares.

## Conclusions

The Pathway Tools Cellular Overview provides a fast zoomable user interface to organism-specific metabolic map diagrams. It provides extensive search capabilities, and will paint experimental datasets onto the metabolic map for visual analysis in a pathway context. This tool can be used from popular browsers (e.g., Firefox, Internet Explorer, Safari, Chrome) without any additional plug-in.

## Availability and Requirements

The Cellular Overview is available at any Web site running the Pathway Tools Web server. The Pathway Tools software is available for free for academic users and for a fee for commercial users. The Cellular Overview can be accessed using a modern Web browser with JavaScript turned on. It has been tested on Internet Explorer, Firefox, Chrome and Safari.

## Authors' contributions

ML participated in the design of the online tool and implemented it. PDK participated in the design of the online tool and managed the project. All authors read and approved the final manuscript.
